# Prevalence of Same-Sex Sexual Behavior in Termites: Persistence Under Mate-Seeking Stress Absence

**DOI:** 10.3390/insects17040400

**Published:** 2026-04-08

**Authors:** Yong-Hui Wang, Huan Wang, Jia Wu, Bei Du, Ya-Lin Xiao, Xin-Yue Li, Ya-Nan Dong

**Affiliations:** 1 College of Medicine, Shaanxi Institute of International Trade and Commerce, Xianyang 712046, China; 2 School of Life Science and Technology, Northwestern Polytechnical University, Xi’an 710129, China; 3 College of Pharmacy, Shaanxi Institute of International Trade and Commerce, Xianyang 712046, China

**Keywords:** behavioral plasticity, same-sex sexual behavior (SSB), sex-specific behavior, tandem, termite

## Abstract

Same-sex sexual behavior is an enigma in behavioral ecology as it does not result in reproduction. Previous studies on the evolution of same-sex sexual behavior have primarily focused on factors such as recognition mechanisms, resource constraints, and challenges in securing an opposite-sex partner, while overlooking the investigation into active same-sex sexual behavior without environmental stress. Here, through experimentation, we investigated the stability of same-sex pairs in tandem running, the mating behavior of pairs that form same-sex tandem runs, and the occurrence of same-sex sexual behavior (SSB) when opposite-sex partners are available. Results indicated that while the stability of same-sex tandems is lower than that of opposite-sex tandems, both males and females are capable of expressing sex-specific behaviors typical of the opposite sex and forming same-sex tandems. Notably, SSB was observed not only in same-sex pairing contexts but also when individuals had the autonomy to choose between same-sex and opposite-sex partners. These results demonstrate that same-sex sexual behavior in termites is not a behavioral response to environmental stress (e.g., the absence of opposite-sex partners) but rather an actively expressed behavior. These findings highlight the role of sex role plasticity in the evolution of same-sex sexual behavior, providing a new mechanistic perspective for understanding same-sex sexual behavior in social insects.

## Simple Summary

The evolution of same-sex sexual behavior (SSB) has often been attributed to environmental constraints, such as a lack of opposite-sex partners. Our study challenges this prevailing assumption by demonstrating that in termites, SSB is an active behavioral expression not driven by partner scarcity or immediate stress. Through controlled experiments, we show that individuals exhibit remarkable sex-role plasticity, engaging in same-sex tandem runs and mating even when opposite-sex partners are available. These findings shift the mechanistic understanding of SSB in social insects from a passive compensatory response to an intrinsic component of behavioral flexibility, influenced by sex-role plasticity. This provides a novel framework for exploring the evolution of complex social behaviors beyond traditional adaptive pressures.

## 1. Introduction

Same-sex sexual behavior (SSB) refers to attempted sexual activity between individuals of the same sex and includes same-sex tandem running and same-sex mating [1,2,3]. The prevalence of SSB has long been considered an evolutionary puzzle, as individuals engaging in SSB invest time and energy in mate-seeking but gain no direct reproductive benefits [1,4,5,6]. In many species, SSB has been attributed to errors in sexual recognition [7,8,9,10]. Beyond recognition errors, SSB may alternatively serve as an adaptive strategy to gain fitness [4,11,12,13]. In termites, this strategy enhances individual fitness by providing critical survival benefits when heterosexual partners are unavailable. Specifically, same-sex pairing can double the escape probability from predators [14] and facilitate cooperative nesting and mutual grooming, which are essential for long-term survival and preventing pathogen infection during the dispersal phase [12]. Thus, SSB acts as a “waiting strategy” that maintains individual viability until a heterosexual mate is encountered. In these two scenarios, a common feature is that individuals in same-sex pairs exhibit behavioral role plasticity. Specifically, one individual can adopt behavioral acts typical of the opposite sex to facilitate stable social interactions. Sex-specific behavior plays an important role in mutual sexual recognition during sexual encounters [15,16]. Therefore, the occurrence and maintenance of SSB may be highly dependent on the “cross-sex borrowing” of sex-specific behaviors (i.e., expressing sex-specific behaviors typical of the opposite sex).

Males and females typically assume distinct roles, manifesting sex-specific behavioral disparities during the reproductive process [17,18,19]. In many termite species, females generally emit mating signals and subsequently select dominant males as partners for copulation [20,21]. Conversely, males act as both competitors and providers, frequently displaying prowess and attractiveness to attention females. They also compete with other males to secure mating privilege with females [17]. Notably, in termites and other social insects with long-term biparental care, the formation of stable opposite-sex bonds also relies on the recognition and response to sex-specific behaviors [22]. If sex roles exhibit flexibility, allowing individuals to display behaviors characteristic of the opposite sex, it is plausible that two males could interact such that one assumes a female role, expressing female-specific traits and thereby precipitating male–male sexual behavior. A similar situation may occur when two females encounter each other. We suggest that these behavioral changes are a byproduct of the high behavioral plasticity evolved for heterosexual coordination. In termites, specific sex roles are essential for tandem running, with females typically leading and males following. However, because pairs frequently separate, individuals must flexibly adjust their behaviors to reunite. Thus, when two individuals meet, they enter a social feedback loop, adjusting their roles to minimize interaction conflict and achieve stable movement. Consequently, the plasticity of sex-specific behavior may increase the incidence of SSB when two same-sex individuals meet each other.

Mate pairing in termites provides an exemplary model system for investigating the evolution of SSB. In the breeding season, large numbers of alates disperse annually from their natal colony to find new nesting sites [20,23,24]. In termites, male–female tandem running is a well-established prelude to pair formation and subsequent mating, serving as a critical behavioral step in the reproductive process. During this period, these sex-specific behaviors are characterized by males consistently following females and maintaining highly coordinated contact while scouting for suitable colony-founding sites [13,22,25,26]. In tandem runs, the individual in front initiating and directing movement was designated the leader, and the individual maintaining close antennal contact while following behind was designated the follower. Generally, the female assumes the role of leader, while male acts as the follower [22]. If the pair becomes accidentally separated during tandem running, the female pauses, while the male continues moving and searching [27,28]. In this scenario, the female takes on a waiting role, while the male acts as the seeker. In this study, we specifically focused on these key components of the behavioral suite-tandem running coordination and mating attempts—as they represent the most prominent manifestations of SSB in this species and provide a measurable framework for analyzing behavioral plasticity. Recent research suggests that termites exhibit plasticity in sex-specific behaviors, wherein females and males can express the behaviors typical of the opposite sex [26]. When two same-sex individuals meet, one of the pairs expresses the behavior of the opposite sex, which is likely to lead to same-sex behavior. Given that neither females nor males possess external sclerotized genitalia, there are no physical impediments to same-sex individuals mating [20,29]. Termites copulate by aligning their abdominal ends, allowing for direct contact between the abdominal tips. This posture does not require intromission of specialized genital structures, enabling both male–male and female–female pairs to engage in copulatory behavior by simply bringing their abdominal ends together in a manner similar to heterosexual pairs (Section 3). Therefore, we speculated that SSB in termites is not a passive consequence of recognition errors or a mere response to the absence of opposite-sex partners but rather an active behavioral expression driven by sex-role plasticity. To test this hypothesis, we formulated the following predictions: (1) same-sex tandem running will occur in both male–male and female–female pairings, where one individual in each pair adopts the behavioral role typical of the opposite sex (because two identical roles cannot achieve coordinated movement); (2) the stability and coordination of same-sex tandems will be comparable to those of heterosexual tandems; and (3) same-sex mating will persist even when opposite-sex partners are available. Crucially, if SSB were merely a transient sex recognition error, these interactions should be brief and quickly abandoned; therefore, we predict its duration will remain significant, indicating active behavioral maintenance rather than a simple mistake. These hypotheses and predictions directly structured our experimental approach.

To substantiate our hypothesis, we rigorously quantified and compared sex-specific behaviors exhibited by *Reticulitermes chinensis* Snyder, specifically the number and duration of tandem running, the speed of tandem runners, and behavioral changes of tandem runners before and after separation, within both same-sex and heterosexual pairings. Furthermore, we examined mating behaviors in same-sex pairings, as well as those displayed when individuals could autonomously choose between a same-sex and an opposite-sex mating partner. These findings will provide new insights into the prevalence of SSB in termites, the sex-specific behavior displayed by individuals, and the environmental stress associated with the absence of opposite-sex partners.

## 2. Materials and Methods

### 2.1. Termite Collection

The three colonies of *Reticulitermes* used in this study were collected from Zibaishan Nature Reserve in Baoji City, Shaanxi Province, China, in 2022, just before the onset of their swarming season. The nests situated within decayed timber were carefully transferred to the laboratory along with the decayed timber, and each was placed in a separate plastic box (dimensions: 70 × 60 × 50 cm), with a fine nylon mesh covering the top. The temperature inside the boxes was regulated at 16 °C until the commencement of the experiments. Prior to each experiment, the plastic boxes were conveyed to an environment adjusted to 30 °C to simulate the climatic conditions of their initial dispersal flight. All experiments involving alates were performed within 24 h after the alates shed their wings. Males and females were distinguished based on the morphology of the 7th abdominal sternite, which is broader in females than in males. This identification was performed under a stereomicroscope before the experiments began (SMZ745; Nikon, Tokyo, Japan).

### 2.2. Experimental Setup

In this study, we established an experimental arena consisting of a 6 cm or 9 cm diameter Petri dish (the size of the Petri dish can be adjusted according to the experimental needs) filled with moistened filter paper, which served as a substrate and moisture source (Petri dish: Jet Biofil, Guangzhou, China; filter paper: NewStar, Hangzhou, China). All observations were conducted within this arena. A high-definition (HD) camera (Nikon D7000 with 60 mm lens, Tokyo, Japan) was positioned 30 cm directly above the arena to record behaviors.

We introduced single tandem pairs of female–female (F-F), male–male (M-M), and female–male (F-M) into the observation arena (6 cm Petri dish), respectively. In each replicate, the two individuals’ thorax were marked with different colors to distinguish them using Street Graffiti paint (using Uni-Paint markers PX-21 Mitsubishi Pencil Company, Tokyo, Japan). Videos were captured at a rate of 25 frames per second for each individual following the resumption of tandem running. Each type of tandem running was replicated 30 times (10 repetitions per colony), with each replicate using different individuals to ensure the independence of observations. We recorded tandem running events over a 3 min (18:00–22:00) period, employing methods described previously [29,30]. A tandem running episode was defined as beginning when one individual followed another at a distance of less than one body length for at least 3 consecutive seconds and ending when the distance exceeded two body lengths for more than 2 s or when one individual stopped moving and the other did not wait/return within 5 s. Trajectories were analyzed using EthoVision XT 15 video-tracking software (Noldus, The Netherlands). To generate automated measurements of behavior, the software was first provided with the test arena dimensions (6 cm) for accurate spatial analysis. In the trial control settings, target termites were selected based on their pre-marked colors, and videos were captured at 5 frames per second. To ensure consistent time settings across recordings, we used the software’s video acquisition system to capture 3 min of video per trial. The distance traveled and walking speed were calculated using the system. Motion trajectories and heatmaps were generated after video capture.

To further investigate whether pairs that form same-sex tandem runs can engage in mating behavior, single pairs of F-F and M-M individuals were introduced into the observation arena (6 cm Petri dish), respectively. Subsequently, 12 h (18:00–06:00) video recordings were conducted to capture the mating behavioral dynamics. Each same-sex pairing type was replicated 24 times (8 repetitions per colony), with F-M pairs serving as a control group (24 repetitions: 8 repetitions per colony). Each trial used different individuals to ensure the independence of observations.

To investigate whether same-sex mating persists when individuals have access to both same-sex and opposite-sex partners, we introduced groups of 5 females and 5 males into a 12 cm diameter arena. Individuals were allowed to freely interact for 24 h, and all mating events were recorded and categorized by pair type. This design provides individuals with a genuine choice among available partners. Males were marked on the abdomen with white (using Uni-Paint markers PX-21, Mitsubishi Pencil Company, Tokyo, Japan). This experiment involved a 24 h (00:00–24:00) duration of video recordings and 6 replicates (2 repetitions per colony). By recording the frequency and relative proportion of female–female (F-F), male–male (M-M), and female–male (F-M) pairing types over a 24 h period, we assessed whether individuals prioritize opposite-sex mating or continue to engage in same-sex sexual interactions when opposite-sex partners are available.

### 2.3. Behavioral Observation

Mating behavior involves a sequence of behavioral acts, including (1) antennal contact and mutual antennation, (2) abdominal alignment where individuals position themselves in opposite directions with their abdomens in contact, (3) genital contact characterized by sustained abdominal coupling, and (4) maintenance of this position for more than 10 s [22]. When tandem running is accidentally disrupted, the leader (typically the female) pauses, while the follower (typically the male) continues moving and searching. This process involves not only the positional order of individuals during tandem running but also sex-specific behaviors (this refers specifically to the sexually dimorphic strategies exhibited during accidental separation). In our study, the “waiting role” (pausing upon separation) is the characteristic strategy of the leader (typically the female), whereas the “seeking role” (active searching) is the characteristic strategy of the follower (typically the male). Thus, we investigated (1) the number and duration of tandem running events among different combinations in 3 min; (2) the behavioral changes (this term describes the transition from synchronized coordinated movement during an active tandem run to the independent separation responses (the aforementioned pausing vs. searching) that occur immediately following a disruption) of tandem runners from coordinated movement to individual responses before and after separation events.

Termites copulate in an opposite abdominal position, joining their abdominal ends. This behavior occurs within the secure nest after pair formation [30]. In this study, we analyzed videos of individual pairs to measure two parameters: the interval from encounter to mating and the frequency of mating events. Additionally, we observed the videos of a group consisting of 5 females and 5 males to investigate the occurrence of SSB in the presence of the opposite-sex individuals and to determine the proportion of SSB among all mating behaviors. Furthermore, we assessed the duration of each mating event to evaluate the mating behavioral stability.

Based on extensive preliminary observations of *R. chinensis*, interactions shorter than 10 s rarely progressed into coordinated tandem runs or successful copulation, whereas events exceeding 10 s consistently involved stable coordinated movement or successful genital coupling. Therefore, any tandem running or mating event lasting more than 10 s was considered valid. If the interval between two consecutive tandem running or mating events was less than 2 s, in this study, the behaviors were recorded as a single event.

### 2.4. Statistical Analysis

All statistical analyses were performed using IBM SPSS Statistics version 19.0. We applied a one-way ANOVA followed by Tukey’s Honest Significant Difference (HSD) test to compare the frequency and duration of tandem running, the interval between encounter of pair members and copulation, the proportion of different copulation types, and the duration of each copulation across various pairing configurations (F-M, F-F, and M-M). Additionally, Welch’s *t*-tests were used to assess the speed differences between leaders and followers during tandem runs and after their inadvertent disruption.

## 3. Results

**Figure 1:**
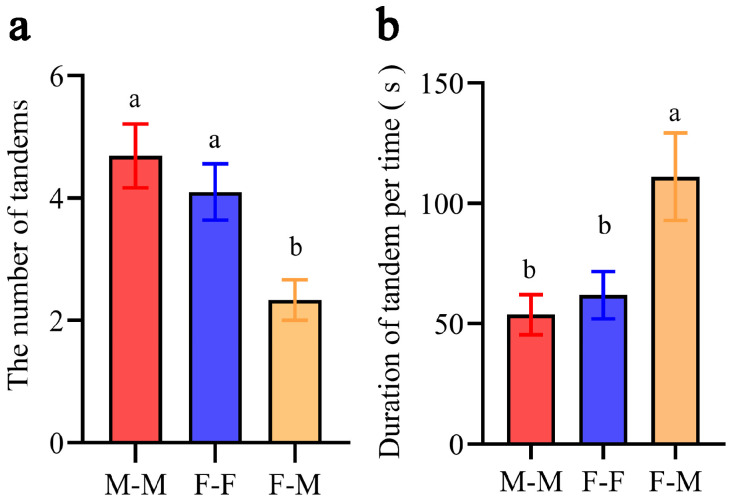
The number and duration of tandem behavior. (**a**) The number of tandems in different pairs. (**b**) The duration of tandem behavior per time. Different letters indicate significant differences at *p* < 0.05.

Not all individuals that encountered each other in the experimental arena formed effective tandem pairs during the observation period. In successfully formed tandem same-sex pairs, the follower consistently follows the leader and maintains highly coordinated movements similar to those in heterosexual (F-M) pairs. Notably, when two females engage in tandem running, the following female exhibits male-typical behaviors (akin to that of the male in F-M tandem running scenario). Similarly, when two males form a tandem running, the leading male shows female-typical behaviors. Both M-M and F-F same-sex tandems occurred more frequently than F-M tandems (Figure 1a; one-way ANOVA: F_2, 29_ = 6.358, *p* = 0.0051; Tukey’s HSD, M-M vs. F-M: *p* = 0.0041; F-F vs. F-M: *p* = 0.048). The duration of each F-M tandem significantly surpassed that of M-M and F-F tandems (Figure 1b; one-way ANOVA: F_2, 107_ = 5.93, *p* = 0.0036; Tukey’s HSD, M-M vs. F-M: *p* = 0.0028; F-F vs. F-M: *p* = 0.024). No significant difference in duration was observed between the two same-sex tandem types (F-F vs. M-M: *p* = 0.92). Although the stability of same-sex tandems is lower than that of opposite-sex tandems, these findings imply that both males and females are capable of adapting their sex-specific behavior and forming same-sex tandems.

**Figure 2:**
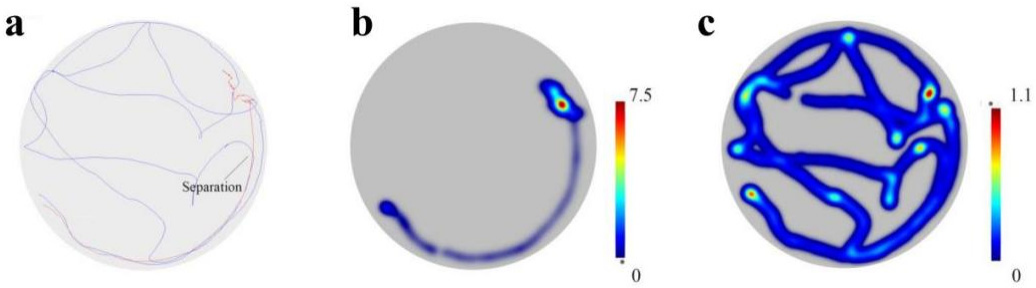
The motion trajectories and heatmaps after tandem-running separation. The dimorphic movement patterns are observed in same-sex pairs, when the tandem became accidentally separated, the leader pauses, while the follower continues to move and search. (**a**) The motion trajectories of leader and follower in same-sex pairs. (**b**) Motion heatmaps of leader. (**c**) Motion heatmaps of follower.

**Figure 3:**
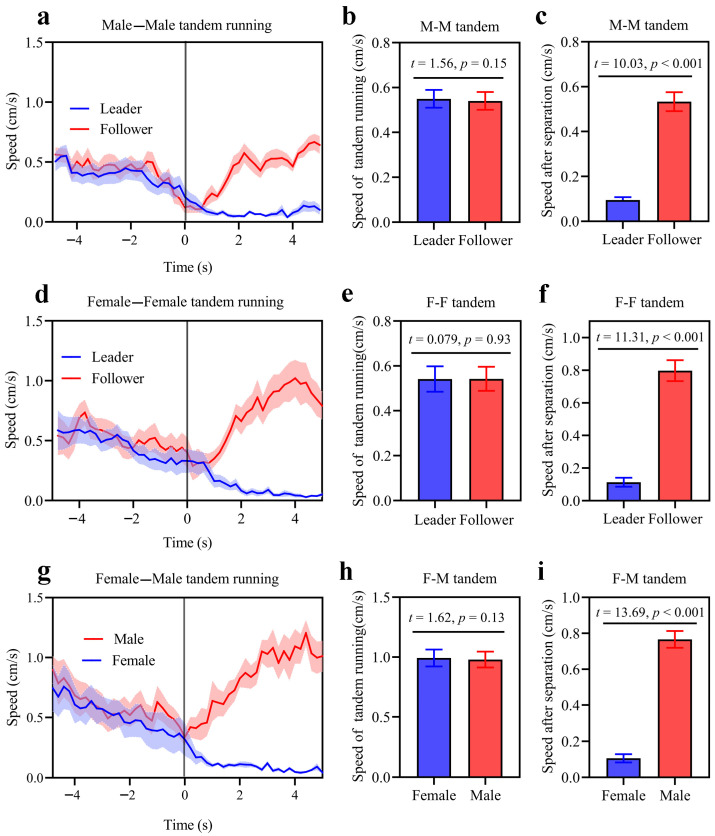
The behavioral change in tandem runners before and after separation events. In same-sex pairings of male (**a**) and female (**d**), the movement patterns of the leader and follower before and after separation mirror those observed in the female and male in a male–female tandem (**g**), respectively. Pair separation takes place at 0 s. The shaded areas represent the mean speed ± standard error. There is no significant disparity in movement velocities between the leader and follower in male–male (**b**), female–female (**e**), and female–male (**h**) pairings. Post-separation, leaders in male–male (**c**) and female–female (**f**) pairs pause, while the followers continue moving; in female–male pairings (**i**), the female pauses while the male remains in motion. The bars denote the mean ± standard error.

Dimorphic movements were also observed in same-sex tandems (when pairs were accidentally separated during tandem running, they showed distinct sexually dimorphic movements, where females paused for long periods while males paused only briefly and moved actively) [22] (Figure 2 and Video S1). During tandem runs, leaders and followers exhibited strongly synchronized movements, and there was no significant difference in the movement speeds of the leader and follower in same-sex M-M (Figure 3a,b: *t*-test, *t* = 1.56, *df* = 11, *p* = 0.15) and F-F (Figure 3d,e: *t*-test, *t* = 0.079, *df* = 11, *p* = 0.93) tandem running. This is similar to the pattern observed in heterosexual (F-M) tandems (Figure 3g,h: *t*-test, *t* = 1.62, *df* = 11, *p* = 0.13). However, when tandem running was accidentally separated, leaders and followers showed distinct movements. In separated M-M same-sex tandems, the follower maintained male-typical behavior characteristic, while the leader displayed the behavior of continuing wait, similar to the female in a heterosexual tandem (Figure 3a,g). In separated F-F same-sex tandems, the leader maintained female-typical behaviors (paused), while the follower displayed continued movement and searching behavior, like the male in a heterosexual tandem (Figure 3d,g). Consequently, the movement speed after separation differed significantly between leaders and followers in M-M (Figure 3c: *t* = 10.03, *df* = 11, *p* < 0.0001), F-F (Figure 3f: *t* = 11.31, *df* = 11, *p* < 0.0001) and F-M tandem running (Figure 3i: *t* = 13.69, *df* = 11, *p* < 0.0001).

**Figure 4:**
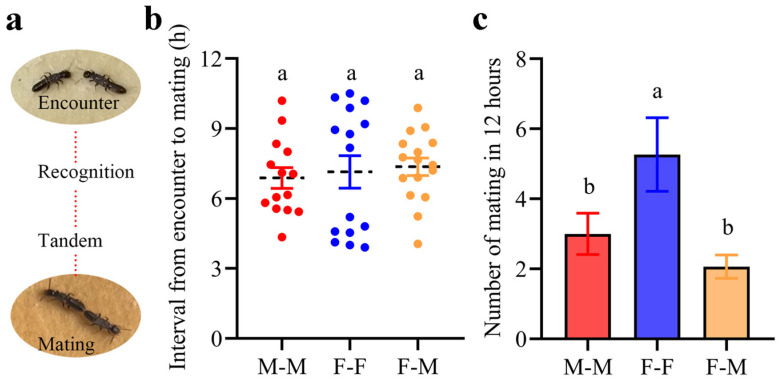
The interval from pair formation to mating and the number of matings of each pair in 12 h. (**a**) The process from encounter to mating and the posture of mating. (**b**) Interval from pair formation to mating. (**c**) Number of matings of each pair in 12 h. Different letters indicate significant differences at *p* < 0.05.

We found that both same-sex and opposite-sex pairs engage in copulation in a similar manner: they align abdominally (abdominal ends joined) (Figure 4a and Videos S2–S4) and copulate multiple times in post-pair formation. Our experimental observations revealed that same-sex mating behaviors were observed as early as 4 h after pair establishment. The time interval between pair formation and the initiation of mating did not significantly differ among F-F pairs, M-M pairs, and F-M pairs (Figure 4b; one-way ANOVA: F_2, 42_ = 0.21, *p* = 0.81). The frequency of mating events within a 12 h period varied significantly among F-F pairs, M-M pairs, and F-M pairs (Figure 4c; one-way ANOVA: F_2, 42_ = 5.42, *p* = 0.008). Specifically, F-F pairs exhibited a significantly higher mating frequency compared to F-M pairs (*p* = 0.007). Conversely, no significant difference in mating frequency was observed between F-M and M-M pairs (*p* = 0.62).

**Figure 5:**
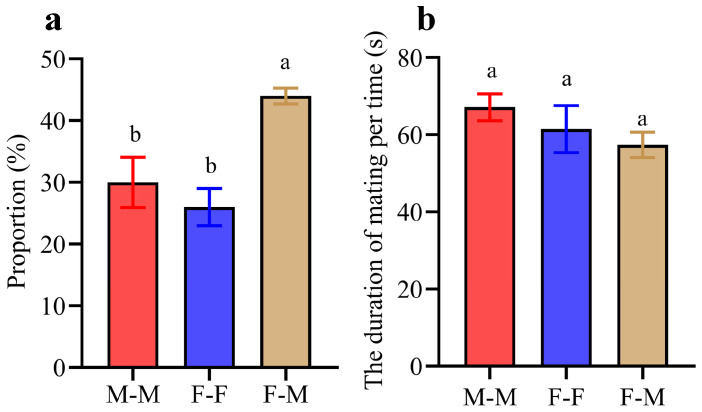
The proportion and duration in different types of mating. Each experimental group consists of 5 females and 5 males. (**a**) The proportion of types of mating. (**b**) The duration of mating in each time. Different letters indicate significant differences at *p* < 0.05.

Even when same-sex and opposite-sex partners were present in the same arena, both female and male individuals engaged in same-sex mating behaviors throughout the observation period. In groups composed of 5 females and 5 males, the distribution of mating behaviors across F-F (Video S2), M-M (Video S3), and F-M (Video S4) pairings exhibited significant disparities (Figure 5a; one-way ANOVA: F_2, 15_ = 9.81, *p* = 0.0055). Specifically, the frequency of F-M mating was significantly higher than that of same-sex mating (Tukey’s HSD, M-M vs. F-M: *p* = 0.023; F-F vs. F-M: *p* = 0.005). Furthermore, the duration of same-sex mating interactions, whether between M-M or F-F pairs, was comparable to that of heterosexual (F-M) mating (Figure 5b; one-way ANOVA: F_2, 67_ = 1.629, *p* = 0.203). In termites, the stable proportion and duration of such behavior confirm that same-sex mating is not a behavioral response to environmental stress that occurs in the absence of opposite-sex partners.

## 4. Discussion

Our study reveals that the sex-specific behaviors of termites exhibit a high degree of flexibility, with both females and males capable of the “cross-sex borrowing” of sex-specific behaviors and forming same-sex tandem runs. In heterosexual tandems, the leader–follower behavioral dynamic is typically determined by sex [22]. Leader females pause to wait if separated from males, whereas follower males engage in extensive searching for their partner upon separation [22,25,27]. However, both females and males retain the behavioral potential to flexibly assume the role of the opposite sex when encountering a same-sex individual: for instance, a female follower in a F-F tandem may exhibit male-typical behaviors, while a male leader in a M-M tandem may display female-typical behaviors. Moreover, in both F-F and M-M same-sex pairs, leader and follower roles were observed to switch across successive tandem bouts, further demonstrating the dynamic nature of sex-role plasticity. Notably, previous investigations have suggested that sex role plasticity is ancestral in termites, not limited to *Reticulitermes* but also present in *Cryptotermes*, *Zootermopsis*, and *Coptotermes* [26]. The expression of sex-specific behaviors typical of the opposite sex during tandem running is indispensable for the successful coordination of same-sex pairs, providing a robust foundation for the evolutionary emergence of SSB.

Tandem running is one of the most pivotal behaviors in the reproductive lifecycle of termites, serving as a key mechanism for mating partner selection [27,31]. It also lays the foundation for subsequent reproductive activities such as nest building, grooming, and mating [29]. After the nuptial flight, the process from the heterosexual encounter to the establishment and maintenance of tandem running involves specific mechanisms, with sex pheromones playing a critical role [32]. Consequently, same-sex pairing has been hypothesized to result from misidentification influenced by sex pheromone signals [5,14] or, alternatively, as an adaptive strategy adopted under environmental pressures (e.g., predators, sex ratio imbalances) to gain fitness benefits [31,33]. However, this explanation fails to account for two key observations: the absence of tandem behavior where males lead and females follow, and the fact that in same-sex tandem, one individual of either sex actively exhibits opposite-sex role behavior. For example, in same-sex female tandems, one female needs to switch its sex role before initiating the tandem runs when not approached by males. The occurrence of M-M tandem running also requires this role switch. These observations suggest that same-sex tandem runs are the result of the active sex-role plasticity of individuals rather than a random behavioral error. It is conceivable that same-sex tandem behavior serves additional, as yet unidentified functions, such as facilitating same-sex colony establishment or sexual behavior. Although our study focused on plasticity in same-sex encounters, it is possible that similar behavioral flexibility could occur in male–female pairs under specific conditions (e.g., when one individual is injured or when demographic factors affect mate availability). Future studies examining the full range of behavioral plasticity across all pairing types would be valuable

Reviewing termite colony lifecycles and reproductive processes, it is noted that mating often occurs within a secure airtight nest following the formation of a pair [20,32]. Directly observing the mating behavior of termites, especially SSB is significantly challenging. Consequently, previous research on termite same-sex behavior has predominantly focused on the more observable phenomenon of same-sex tandem running [12,31] and how same-sex pairings provide survival benefits [13,34,35]. In this study, we found that both same-sex and/or heterosexual pairs appear to complete the process from pair formation to mating in a relatively short time. Within colonies, mating is characterized by multiple occurrences, with the majority occurring within 24 h of pair formation. This result is similar to what we observed in *R. aculabialis* and *R. flaviceps* [27]. This result suggests that same-sex mating behavior readily occurs when the opposite-sex individuals are absent. However, in different species, there may be differences in the time interval from pair formation to mating and mating frequency. This difference may vary depending on the species’ biological characteristics, as well as the time interval between alates shedding their wings and experimental pairing. Regardless of this interval, the relatively high frequency of SSB further indicates that SSB is common in termites.

In the fields of biology and ethology, same-sex pairing and mating behaviors are complex phenomena, potentially influenced by multiple factors including genetics [36,37], social interactions [4,38], learning [4,39], limited resources, and challenges in securing an opposite-sex partner [12,40,41]. Thus, the mating behaviors observed in our same-sex pairing experiments may be a forced reproductive strategy exclusively in the absence of an opposite-sex partner. However, our findings indicate that even when individuals of both sexes are concurrently present in the same arena, SSB persists in termites. The higher frequency of heterosexual mating also further indicates that termite mating behavior in mixed-sex environments is not indiscriminate [1]. This observation confirms that the SSB exhibited by termites is not exclusively attributable to the absence of the opposite sex.

Additionally, previous studies have revealed that same-sex tandem behavior is not merely a strategy to reduce predation risks [31] or a reproductive tactic when opposite-sex partners are absent; it may also function as preparation for same-sex mating. Notably, same-sex behavior in termites may even exist independently of environmental stress. This is a natural phenomenon observed in other species, such as same-sex behavior in female bonobos [3,42], Laysan albatrosses [43], and penguins [44]. Unlike stress-induced responses, same-sex behavior in these species is linked to biological, social, or preferential factors. These findings collectively suggest that same-sex behavior can be a natural component of an animal’s social or reproductive behavioral repertoire, rather than merely a “response to stress”.

Furthermore, a primary limitation of this study is the difficulty in precisely quantifying real-time mate preferences during the initiation of tandem runs. While our 5 × 5 group assays provided a realistic ecological context, the complex interactions and frequent partner switching within these groups made isolating individual decision-making highly challenging. To address this, we propose a simplified ‘triad’ experimental model (e.g., 2 females and 1 male, or vice versa) as a key direction for future research. By reducing the number of individuals, this model will enable the high-resolution tracking of tandem duration, partner-switching frequency, and individual decision-making processes. This approach will yield more granular data to distinguish whether same-sex behavior (SSB) is a stochastic product of high-density interactions or a strategic manifestation of behavioral plasticity under direct social competition. Due to the strict seasonality of termite alate dispersal, this investigation is planned for the next swarming season to further deepen the mechanistic insights gained in this study.

## Data Availability

The original contributions presented in this study are included in the article/Supplementary Materials. Further inquiries can be directed to the corresponding authors.

## Supplementary Materials

The following supporting information can be downloaded at https://www.mdpi.com/article/10.3390/insects17040400/s1, Video S1: Dimorphic movements in same-sex tandem; Video S2: Female–Female mating in groups composed of 5 females and 5 males; Video S3: Male–Male mating in groups composed of 5 females and 5 males; Video S4: Female–Male mating in groups composed of 5 females and 5 males.
